# Anaphylaxis severity grade, during oral food challenges, assessed by five different classifications

**DOI:** 10.1111/pai.70065

**Published:** 2025-03-21

**Authors:** Yanis Bouderbala, Evangéline Clark, Luciana Kase Tanno, Pascal Demoly, Davide Caimmi

**Affiliations:** ^1^ Allergy Unit Hôpital Arnaud de Villeneuve, University Hospital of Montpellier Montpellier France; ^2^ Allergy Unit Hospital of Mont de Marsan Mont‐de‐Marsan France; ^3^ IDESP, UMR 1318 University of Montpellier – INSERM Montpellier France; ^4^ WHO Collaborating Centre on Scientific Classification Support Montpellier France

**Keywords:** adrenaline, anaphylaxis, classifications, ICD‐11, severity

## Abstract

**Background:**

While the definition of anaphylaxis is clear, its grade of severity remains a subject of debate. The objective of this study was to evaluate the possible discrepancies in the severity scoring system for anaphylaxis in patients with a positive food challenge (OFC), differentiating anaphylactic and non‐anaphylactic reactions, using the WHO for the 11th version of the International Classification of Diseases (ICD‐11) as the main reference.

**Methods:**

We conducted a retrospective observational study at the University Hospital of Montpellier, France, including patients with a positive food OFC between 2018 and 2022. We classified the severity of each reaction based on 5 different classifications. We also compared patients presenting an anaphylactic versus a non‐anaphylactic reaction during the OFC in terms of symptoms and therapeutic approach.

**Results:**

235 patients presented a positive OFC between January 2018 and December 2022: 143 (60.9%) suffered from anaphylaxis, according to the ICD‐11 classification. When comparing the different classifications, a complete concordance was recorded in 8 patients (5.6%) only. All classifications showed a good sensitivity (99.3%–100%), but different specificity (67.4%–93.5%), and discrepancies between them were shown in most patients. Respiratory and gastrointestinal symptoms were significantly more frequent in the anaphylaxis group. Adrenaline was injected in only 47.6% of patients suffering from anaphylaxis, even in a specialized setting.

**Conclusion:**

Our work highlights the need to refine the different scoring systems and, even better, to disseminate unified diagnostic criteria, such as the ICD‐11 ones, to avoid the underdiagnosis of anaphylactic reactions and ensure appropriate management for all allergic patients.

**Key message**

Physicians need to easily communicate between them on anaphylaxis, without risking misunderstanding the severity of a reaction. The multiple existing classifications do not allow a univocal grading of the severity of anaphylaxis. An easy approach is needed, as the one proposed by the WHO, which will also be used by non‐specialists to classify anaphylactic reactions.

## 1 INTRODUCTION

Anaphylaxis is the most severe immediate hypersensitivity reaction, which may potentially result in death.^1^ In 2013, Panesar reported an incidence of anaphylaxis between 1.5 and 7.9 per million inhabitants per year.^2^ Although the number of deaths due to anaphylaxis remains low, the frequency of anaphylactic cases and of cases at risk of anaphylaxis has been increasing in recent years.^3^

The clinical definition of anaphylaxis was initially described by Ring and Messmer in 1977.^4^ A recent definition is that anaphylaxis is a serious systemic hypersensitivity reaction that is usually rapid in onset and may cause death.^5^, ^6^ The World Allergy Organization (WAO) anaphylaxis committee^5^, ^6^ proposes, for the diagnosis, the assessment of at least one of these two criteria:

- An acute onset of an illness (minutes to several hours) with involvement of the skin, mucosal tissue, or both, associated with a respiratory compromise and/or a reduced blood pressure (or symptoms of end‐organ dysfunction), and/or severe gastrointestinal symptoms;
- An acute onset of hypotension or bronchospasm, or laryngeal involvement in an individual with a previous allergy and a suspected exposure to this allergen.

As for the classification of the severity of an anaphylactic reaction, such a topic remains a matter of debate. In recent years, several classifications emerged, enriching the existing ones, such as those from the ICD‐11,^7^ the CoFAR one,^8^ or those by Dribin,^9^ the EAACI (European Academy of Allergy and Clinical Immunology),^10^ and Blazowski,^11^ and providing different scores to grade the severity of an allergic anaphylactic reaction.

These different classifications allowed a major advancement in the management of anaphylaxis (guiding the early use of adrenaline); however, differences between them perpetuate a lack of uniformity in grading different levels of severity and, consequently, potential differences in the therapeutic approach to patients experiencing the reaction. These differences in the proposed classifications also depend on allergenic triggers, age groups, and on the fact that the reaction may occur during a *real*‐*life* exposure or in a hospital iatrogenic setting. In clinical practice, the proliferation of classifications may be a limit when comparing epidemiological studies and trying to determine risk factors of the most severe cases. Moreover, it led to a lack of reproducibility, as the same reaction may not receive the same score from different classifications, which may then impact both the timing and the choice of a specific treatment.

The primary objective of this study was to evaluate the possible discrepancies in the severity scoring system for anaphylaxis by applying it in patients who presented a positive food challenge in the Allergy Unit of a University Hospital, using the WHO for the 11th version of the International Classification of Diseases (ICD‐11) as the main reference.

## 2 METHODS

We conducted a retrospective observational monocentric study at the Allergy Department of the University Hospital of Montpellier, France. We included all patients—adults and children—who underwent a positive oral food challenge (OFC) for an IgE‐mediated food allergy between January 2018 and December 2022. Open OFCs were performed following PRACTALL recommendations.^12^, ^13^ All included food challenges were performed to either confirm or exclude a possible food allergy in patients having presented a previous clinical reaction possibly due to the tested food or put on an elimination diet based on the results of a previous allergy work‐up. All those who presented a history of anaphylaxis had a prescription for Adrenaline Auto‐Injector (AAI) and patients and/or caregivers are required to present with their emergency kit (including AAI) the day of the OFC.

The study was approved by the Institutional Review Board of Montpellier (IRB‐MTP_2022_09_202201201).

Patients' characteristics and information were extracted from the Food Allergy and Hypersensitivity Database® (FAHD®). This database includes all information on patients' general information, clinical history, comorbidities, possible long‐term treatment, results of performed in vivo and in vitro tests, doses administered during the food challenge, clinical symptoms appearing during the challenge, possible acutely administered treatment, and follow‐up of the patients after the challenge. In case of missing data, a manual extraction was performed from the patients' electronic and/or paper medical records. For each patient, the following data were collected: age, sex, clinical manifestations experienced during the reported clinical history, symptoms presented during the OFC, severity of the reaction according to different classifications, and administered treatment. As for those patients having experienced multiple allergic reactions to a specific food, we chose to include information on the most severe experienced one.

We manually reclassified the severity of all reactions according to 5 classifications of food allergy‐related anaphylaxis published in the literature (International Classification of Diseases, 11th version, ICD‐11; CoFAR; Dribin; EAACI; Blazowski).^7^, ^8^, ^9^, ^10^, ^11^ These classifications are shown in Table S1. We chose to use the ICD‐11 classification^7^ as the reference one. Two independent allergists classified the severity of each reaction. Any disagreement between the two allergists was resolved through discussion with a third specialist to reach a final consensus.

The primary objective of the study was to evaluate the grading of a reaction considering the different classifications. Secondary objectives included the assessment of symptoms presented by patients during a positive OFC, compared with those reported in the patients' initial clinical history, and the evaluation of the appropriateness of adrenaline injection, according to the severity of the symptoms and based on the guidelines of the European Academy of Allergy and Clinical Immunology (EAACI),^14^ by also comparing data in the group of patients experiencing anaphylactic reactions with those not suffering from anaphylaxis during the challenge.

Qualitative variables were evaluated as frequencies and percentages, and the quantitative variables were evaluated as median and interquartile range. Spearman's correlation coefficient was used to examine the correlation between the different classifications, and the gold standard (ICD‐11 classification). ROC (Receiver Operating Characteristic) curves were plotted, with sensitivity, specificity, area under the curve (AUC), and corresponding optimal thresholds used to evaluate the diagnostic performance of each classification. AUCs were compared by the DeLong test. comparisons of the qualitative data between treated and untreated with epinephrine/adrenaline patient groups were carried out using chi‐square or Fisher's exact test for small samples. Differences between the anaphylaxis and the non‐anaphylaxis groups (general data, symptoms, and administered treatments) were assessed with the Wilcoxon rank‐sum test since they were not normally distributed. Data were considered statistically different if the *p*‐value was ≤.001. All analyses were performed using SAS version 9.4 (SAS Institute Inc., Cary, NC, USA).

## 3 RESULTS

**FIGURE 1:**
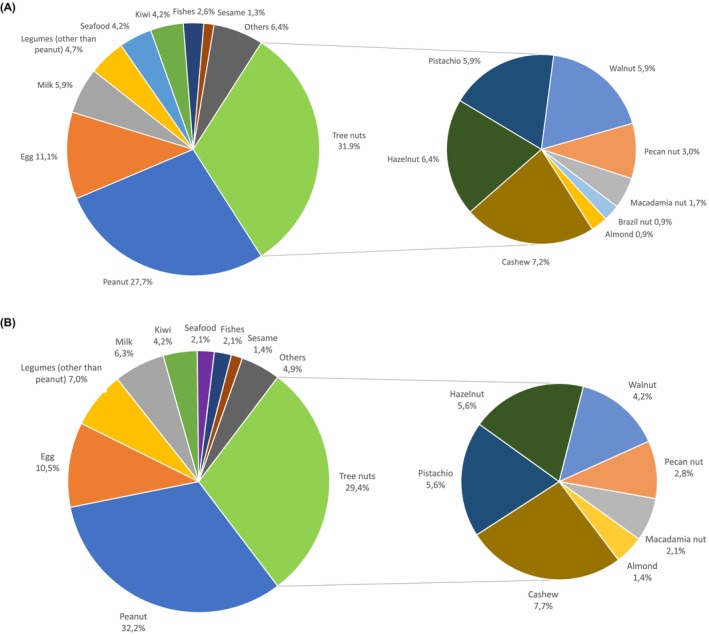
Distribution of foods causing any allergic reactions in our group of 235 patients (A) and in the subgroup of 143 anaphylactic patients (B).

235 patients presented a positive OFC between January 2018 and December 2022: 143 (60.9%) suffered from anaphylaxis, according to the ICD‐11 classification. The median age was 9 years both in the whole population (25th–75th: 6–14; min‐max: 1–62) and in the subgroup of 143 patients with anaphylaxis (25th‐75th: 6–13.5; min‐max: 2–61) (Table 1). In the whole group of patients with a positive OFC, 83 (35.3%) had no initial medical history with the tested food. When considering the most frequent food responsible for a positive OFC, the main group includes tree nuts (31.9%), followed by peanut (27.7%) and eggs (11.1%) (Figure 1A). As for tree nuts (Figure 1A), cashew nut were the most common one responsible for allergies (22.7% of this subgroup and 7.2% of the whole cohort).

**TABLE 1:** Characteristics of the included population.

	Anaphylaxis	Non‐anaphylaxis	*p*‐value
	*N* = 143	*N* = 92	
Sex, males, *n* (%)	91 (63.6)	59 (64.1)	.94
Age (year), median (Q1–Q3)	9 (6–13.5)	9.5 (7–14)	.90
Previous/Initial clinical history, *n* (%)			
Anaphylaxis	45 (31.5)	16 (17.4)	.**02**
Skin and mucosa			
Urticaria	56 (39.2)	17 (18.5)	.**001**
Generalized oedema	47 (32.9)	31 (33.7)	.90
Pruritus	11 (7.7)	9 (9.8)	.58
Sneezing	0 (0)	1 (1.1)	.39
Rhinitis	6 (4.2)	2 (2.2)	.49
Conjunctivitis	1 (0.7)	0 (0)	1
Respiratory symptoms			
Oedema of larynx	5 (3.5)	2 (2.2)	.71
Cough and/or bronchospasm	19 (13.3)	5 (5.4)	.**05**
Dyspnea	5 (3.5)	5 (5.4)	.52
Dysphonia	4 (2.8)	0 (0)	.16
Gastrointestinal symptoms			
Abdominal pain	16 (11.2)	9 (9.8)	.73
Vomiting	23 (16.1)	7 (7.6)	.06
Nausea	3 (2.1)	2 (2.2)	1
Cardiovascular symptoms			
Hypotension	4 (2.8)	2 (2.2)	1
Asthenia	0 (0)	2 (2.2)	.15
Tachycardia	1 (0.7)	0 (0)	1
Clinical reaction during oral food challenge, *n* (%)			
Anaphylaxis	143 (100)	0 (0)	
Skin and mucosa			
Urticaria	70 (49.0)	37 (40.2)	.19
Generalized oedema	19 (13.3)	14 (15.2)	.68
Localized Pruritus	43 (30.1)	39 (42.4)	.**05**
Generalized Pruritus	3 (2.1)	1 (1.1)	1
Sneezing	2 (1.4)	0 (0)	.52
Rhinitis	56 (39.2)	29 (31.5)	.23
Conjunctivitis	8 (5.6)	10 (10.9)	.14
Respiratory symptoms			
Oedema of larynx	3 (2.1)	0 (0)	.28
Cough and/or bronchospasm	47 (32.9)	3 (3.3)	<.**001**
Dyspnea	10 (7.0)	0 (0)	.**01**
Wheezing	11 (7.7)	2 (2.2)	.07
Dysphonia	2 (1.4)	0 (0)	.52
Gastrointestinal symptoms			
Abdominal pain	87 (60.8)	28 (30.4)	<.**001**
Vomiting	54 (37.8)	4 (4.3)	<.**001**
Nausea	56 (39.2)	5 (5.4)	<.**001**
Diarrhea	6 (4.2)	1 (1.1)	.25
Gastrointestinal symptoms			
Hypotension	17 (11.9)	0 (0)	.**001**
Asthenia	6 (4.2)	0 (0)	.08
Tachycardia	5 (3.5)	0 (0)	.16

*Note:* Bold values are statistically significant.

### 3.1 Classification of the anaphylactic and nonanaphylactic reactions

In the group of 143 patients who experienced anaphylaxis during the OFC, 109 patients (76.2%) were classified as grade 2 according to the ICD‐11 classification, and 34 patients (23.8%) were classified as grade 3. The distribution of patients according to the severity of the reaction and differentiating between anaphylactic and non‐anaphylactic reactions, based on the different classifications, is shown in Table 2.

**TABLE 2:** Distribution of anaphylactic and non‐anaphylactic patients, following the 5 assessed classifications based on the severity of symptoms.

	Anaphylaxis	Non anaphylaxis		Anaphylaxis	Non anaphylaxis
	*N* = 143	*N* = 92		*N* = 143	*N* = 92
ICD‐11 classification,^7^ *n* (%)					
Grade I	0 (0)	92 (100)			
Grade II	109 (76.2)	0 (0)			
Grade III	34 (23.8)	0 (0)			
Grade IV	0 (0)	0 (0)			
CoFAR classification,^8^ *n* (%)			EAACI classification,^10^ *n* (%)		
Grade I	1 (0.7)	62 (67.4)	Grade I	74 (51.7)	87 (94.6)
Grade II	97 (67.8)	27 (29.3)	Grade II	54 (37.8)	5 (5.4)
Grade III	28 (19.6)	3 (3.3)	Grade III	15 (10.5)	0 (0)
Grade IV	17 (11.9)	0 (0)			
Grade V	0 (0)	0 (0)			
Dribin's classification,^9^ *n* (%)			Blazowski's classification,^11^ *n* (%)		
Grade I	52 (36.4)	81 (88.0)	Grade I	65 (45.5)	86 (93.5)
Grade II	45 (31.5)	8 (8.7)	Grade II	58 (40.6)	6 (6.5)
Grade III	33 (23.1)	3 (3.3)	Grade III	7 (4.9)	0 (0)
Grade IV	13 (9.1)	0 (0)	Grade IV	13 (9.1)	0 (0)
Grade V	0 (0)	0 (0)			

When comparing the different classifications, a complete concordance between all 5 of them, as for severity grading, was recorded in 8 patients (5.6%) only. No patient was classified as presenting a Grade 5 reaction, as possibly proposed by the CoFAR and Dribin's classification. Differences between classifications are shown in the Table S2.

The sensitivity and specificity of the different classifications for identifying anaphylaxis are shown in Table 3 sensitivity resulted in the best for the classification by the EAACI, Dribin, and Blazowski (100%), while specificity was the best for the one by the EAACI (93.5%).

The determination of the area under the curve (AUC) showed that all classifications had a good ability to detect anaphylaxis. In the ROC analysis, the AUCs of CoFAR, EAACI, Blazowski's and Dribin's classifications were 0.83, 0.97, 0.95, and 0.93, respectively. The DeLong test showed a significant difference between the AUCs of the ICD‐11 classification and those of CoFAR (*p*‐value .0001), EAACI (*p*‐value .0117) and Dribin (*p*‐value .001) classifications.

**TABLE 3:** Sensitivity and specificity of the evaluated classification when using the ICD‐11^7^ as the reference one.

Classification	Sensitivity		Specificity	
CoFAR^8^	99.3%	95% CI: 0.97–1.00	67.4%	95% CI: 0.57–0.76
Dribin^9^	100%	95% CI: 1.00	85.9%	95% CI: 0.78–0.92
EAACI^10^	100%	95% CI: 1.00	93.5%	95% CI: 0.78–0.92
Blazowski^11^	100%	95% CI: 1.00	91.3%	95% CI: 0.85–0.97

Abbreviation: CI, confidence interval.

### 3.2 Differences between patients experiencing anaphylactic and nonanaphylactic reactions

Considering the group of 143 patients who presented an anaphylactic reaction during the OFC, 45 of them (31.5%) already had a history of anaphylaxis to the tested food, while 49 (34.3%) had been avoiding the allergen, without having presented a previous reaction. In the group of 92 patients with positive OFC, but without anaphylactic symptoms, 16 (17.4%) had a history of anaphylaxis for the same allergen (*p*‐value <.02). 102 patients (71.3%) presented a more severe reaction during the OFC, if compared to the one recorded in their clinical history. This group includes 98 patients transitioning from grade 1 to anaphylaxis, and 4 transitioning from anaphylaxis to anaphylactic shock.

In patients' clinical history, urticaria was reported by 56 patients in the anaphylaxis group (39.2%) as being present during the initial reaction to the food, compared to only 17 patients in the non‐anaphylaxis group (18.5%) (*p*‐value <.0008). During the OFC, the most frequently presented symptom in the anaphylaxis group was abdominal pain (60.8%), followed by urticaria (49.0%). Other patients' characteristics are shown in Table 1.

Most patients with anaphylaxis were treated with oral antihistamines (93.7%) and oral corticosteroids (69.2%). Inhaled salbutamol was administered in 25.2% of the anaphylactic patients. Adrenaline intramuscular injection was administered only in 47.6% of them (Table 4). Three patients needed two injections of adrenaline: two of them reacted to pecan nut, and one to kiwi. As expected, there was a significant difference between the anaphylactic and non‐anaphylactic groups, as for adrenaline injection (47.6% vs. 4.3%, *p*‐value >.001), but also for oral glucocorticoids administration (68.5% vs. 33.7%, *p*‐value <.001).

**TABLE 4:** Treatment administered upon reaction, during the oral food challenge, in the 235 patients.

	Anaphylaxis	Non anaphylaxis	*p*‐Value
	*N* = 143	*N* = 92	
Intramuscular adrenaline	68 (47.6)	4 (4.3)	**<.001**
Oral antihistamines	135 (94.4)	74 (80.4)	.001
Oral glucocorticoids	98 (68.5)	31 (33.7)	**<.001**
Inhaled salbutamol	36 (25.2)	8 (8.7)	.002
Antihistamine eye‐drops	2 (1.4)	8 (8.7)	.02

*Note:* Bold values are statistically significant.

**FIGURE 2:**
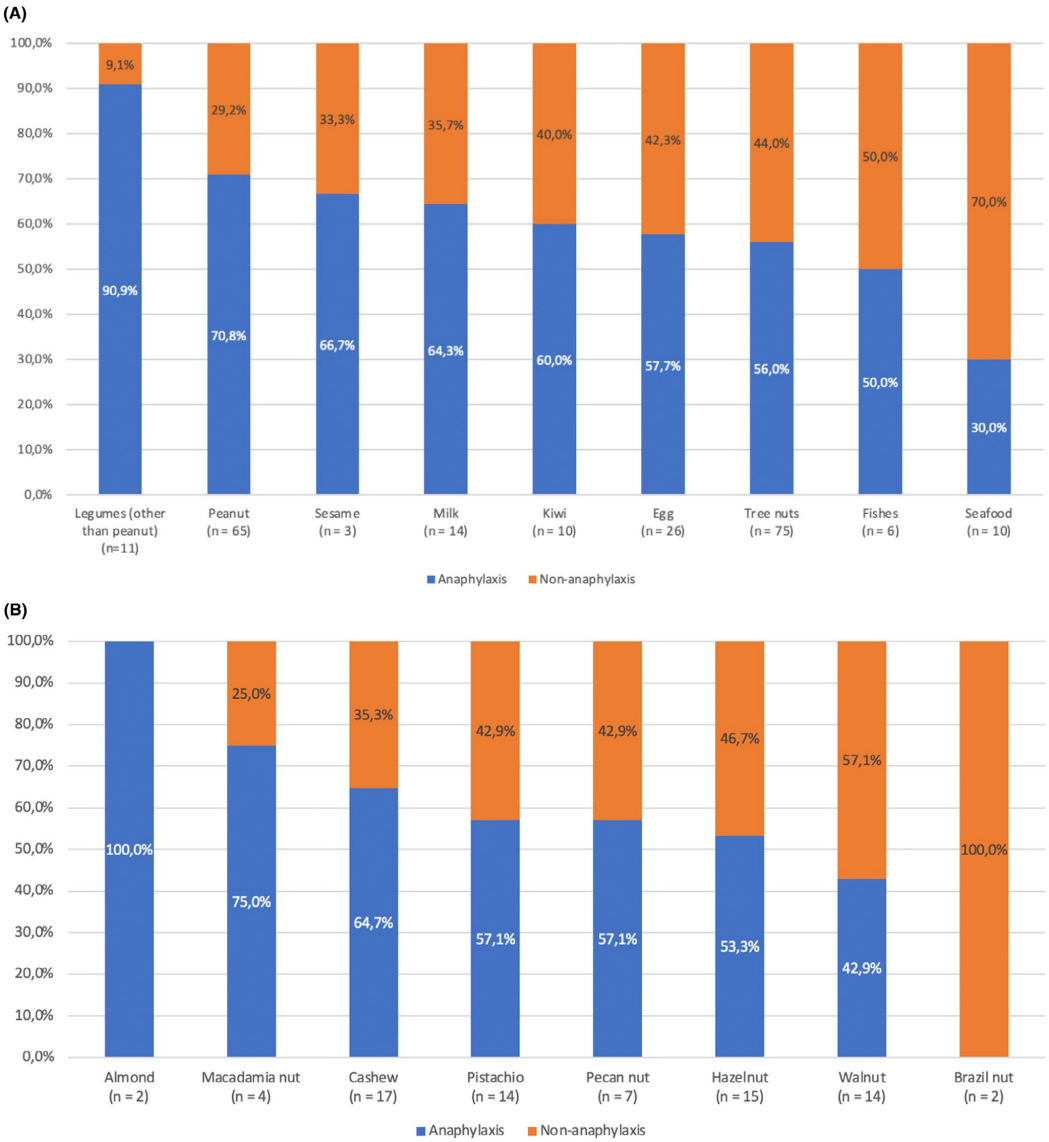
Distribution of anaphylactic and non‐anaphylactic reactions recorded during the oral challenge, with the main tested foods (A) and with tree nuts (B).

As for the most frequent food responsible for an anaphylactic reaction during the OFC, peanuts were responsible for 32.3% of the anaphylactic reactions, followed by tree nuts (29.4%) and by eggs (10.5%) (Figure 1B). As for tree nuts, cashew nut were the most commonly responsible one for anaphylaxis, as already recorded in the whole population (27.5% of this subgroup and 7.7% of the whole anaphylaxis group) (Figure 1B). When comparing anaphylactic and non‐anaphylactic reactions specific to foods (Figure 2), we found legumes (besides peanut) to be more frequently associated with anaphylactic reactions compared to non‐anaphylactic ones (90.9% vs. 9.1%). Patients with a positive OFC to seafood, excluding fish, reacted to the challenge mainly without experiencing anaphylaxis (30.0% vs. 70.0%). Peanuts were responsible for challenge‐driven anaphylaxis in 70.8% of cases. As for tree nuts, almonds triggered anaphylaxis in every positive OFC; on the other hand, patients positive for Brazil nuts never experienced an anaphylactic reaction during the challenge (Figure 2).

## 4 DISCUSSION

Anaphylaxis management and severity labeling is still a subject of debate in 2024, even within a specialized allergy department. The lack of a unanimous international consensus and the multiplicity of published classifications complicate the management of patients in an emergency setting. To our knowledge, this study is the first one comparing different classifications of anaphylaxis and severity levels by using the WHO classification proposed for the ICD‐11^7^ as the reference one. The results of this study demonstrate a certain degree of variability in anaphylaxis classification systems and, therefore, their implications for treatment management, and underline the need to disseminate unified diagnostic criteria for anaphylaxis.

Given the multiple definitions of anaphylaxis, several groups proposed severity scores, often based on expert opinions, but only a few of them have been validated.^15^, ^16^, ^17^, ^18^, ^19^, ^20^ In our study, each one of the 4 assessed classifications provided different degrees of severity when compared with the ICD‐11 one. It is clear that the grading values differ depending on the evaluated classification: indeed, the EAACI's classification^10^ has 3 levels of severity, Blazowski's one^11^ has 4, while those from CoFAR^8^ and Dribin^9^ go from 1 to 5. In his paper, Blazowski already highlighted the discrepancy between several classifications, some of them not necessarily used to classify food‐induced anaphylaxis, and he underlined that a new severity grading system was needed, especially to harmonize the definition of severity and to avoid any delay in the administration of adrenaline.^11^ Also, in a paper by Eller et al., 23 different instruments to classify the severity of an anaphylactic reaction were compared. The article focused on both food‐ and drug‐driven anaphylaxis and highlighted the variety of distributions of severity between the different instruments, especially when considering that grading systems may go from a maximum of 3 to a maximum of 5, making it difficult to compare the currently available classifications between them.^18^ Such an aspect has been clearly demonstrated even in our work, especially when looking at the results presented both in Table 2 and in Table S2. Today, depending on the used classification, it is impossible to simply state the grade of severity of an anaphylactic reaction without contextualizing it to the referred scoring method.

Our work underlines, therefore, the importance of disseminating and using a simple and unified global classification to improve education, clinical approaches to patients, and clear interactions between physicians. We decided to refer to the ICD‐11 classification as a gold standard since it's the one used on a daily base in our unit, and it's easy to use; it is also the classification used by the WHO post‐coding system, which is already used and will be even more implemented over the coming years, being available in all countries for all healthcare professionals and will therefore allow identification of even cases coded by non‐allergists, especially primary care physicians. In any case, current classifications are especially useful for epidemiological and research purposes, but we proved that they're not easy to use in clinical practice, not only by non‐specialized physicians, but even by allergists when they need to promptly treat an allergic reaction during an OFC. We showed that the likelihood of agreement between the different classifications is low, especially if we consider that they are based on 3–5 different grades: such discrepancy shows that the multitude of classifications doesn't help understand the severity of the reaction. Therefore, from an educational point of view, and for a better management between teams and an easier comprehension from non‐specialized physicians (such as emergency doctors), the use of an easy‐to‐understand simple and common classification would help reduce possible misunderstandings and improve the therapeutic approach towards patients.

What still needs to be the cornerstone of the management of anaphylaxis is the injection of IM adrenaline, and the diagnosis of anaphylaxis should remain essentially based on a clinical assessment of the patient. If, on one hand, the injection of adrenaline should never be delayed, regardless of the supposed severity of the anaphylactic reaction, an easy and practical overall accepted classification could help avoid the underuse of adrenaline.

Our study is based on oral food challenge results during which vomiting is a common symptom, often combined with other symptomatic elements warranting the injection of adrenaline.^21^ The most frequently reported symptom during OFC in our anaphylactic patients was abdominal pain, underlying the relevance of gastrointestinal symptoms during food allergy reactions. This result is noteworthy considering that, in some countries such as the UK and Australia,^22^ abdominal symptoms are not considered in the classification of food allergy reactions. On the other hand, the recently published WAO grading system for systemic allergic reactions includes gastrointestinal symptoms at any grade of severity, underlying their relevance in allergic patients.^23^

Indeed, the results of our study show that abdominal pain is often the first symptom found in patients further developing anaphylactic reactions. Therefore, the question arises as to whether this reported symptom should be considered a red flag for a possible anaphylactic reaction.

In our group of patients, 60.9% of patients with a positive OFC reacted with an anaphylactic reaction. Also, ninety‐eight patients (41.7%) developed anaphylaxis during the oral provocation test, while none of them had a clinical history of severe reaction. Main foods associated with a worsening of the reaction, compared with the one reported in patients' clinical history, were peanut (28.8% of patients), eggs (12.0%), and pistachio (8.0%). On the other hand, considering patients with a history of anaphylaxis, 45 (19.5%) presented an anaphylactic reaction during the OFC, while 16 (7%) did not. These data also underline the fact that the evolution of a food allergy remains unpredictable, and OFC should be performed only by well‐trained professionals in a safe hospital environment.

Regarding the management of patients, we recorded a statistical difference in the use of corticosteroids during an anaphylactic reaction. Even though such a therapeutic approach is not recommended,^5^ we could speculate that the fear of not providing enough treatments wrongly pushed the physicians into including glucocorticoids in the list of the administered ones. As for adrenaline administration, our study revealed a delay, or even an absence, in the use of this drug in cases of anaphylaxis, regardless of the classification used by the physician. Similar results were also published by our group when considering data from a French pediatric emergency unit.^24^ A study by Eller et al.^25^ emphasizes the existence of different severity grading systems for anaphylaxis and the use of adrenaline, leading to variations in its administration if based on the grading system. There are no universally validated diagnostic tools to determine which symptoms warrant adrenaline treatment compared to those that do not,^26^ which may explain the underuse of adrenaline found in our study. It should also be underlined that no study proved in a randomized controlled trial that the use of adrenaline is always essential in treating allergic anaphylactic reactions, and that not only more than half of anaphylaxis reactions resolve spontaneously without its injection.^27^ At the same time, clinicians should be aware that the clinical evolution of an allergic reaction after the appearance of the first symptoms is unpredictable, and a close follow‐up of patients is needed to assess the possible secondary progression towards anaphylaxis.^27^

Our study presents some limitations: it is a retrospective study, and some data may therefore be missing from the medical records of certain patients. Also, the choice to use the ICD‐11 classification as the reference one, well justified for its ease of use in daily practice and its validation by the WHO as stated above, could potentially introduce biases. Nevertheless, we also present results that show a certain strength, considering the number of included patients, the double‐blind verification of the classifications by two specialized allergists, and the use of four recent classifications for comparison purposes. We are aware that our data are not representative of the general allergic population facing a reaction in their everyday life. Nevertheless, we believe that our study may be a model to be validated by other institutions since the ICD‐11 is under implementation worldwide.

While the emergency of different classifications of anaphylaxis and of its severity is a real asset for clinical management, their multiplicity creates confusion among healthcare professionals. Therefore, it would be important to consider consolidating these different classifications into one that is both appropriate and intuitive, favoring sensitivity with a good compromise regarding specificity. Our work highlights the need to adopt a universal, intuitive, and easy‐to‐use classification, such as the ICD‐11 one, while destigmatizing at the same time the use of adrenaline.

## AUTHOR CONTRIBUTIONS

**Yanis Bouderbala:** Investigation; writing – original draft; formal analysis; data curation. **Evangéline Clark:** Writing – original draft; formal analysis; data curation; methodology. **Luciana Kase Tanno:** Validation; writing – review and editing; resources. **Pascal Demoly:** Writing – review and editing; validation; supervision. **Davide Caimmi:** Conceptualization; methodology; validation; writing – review and editing; project administration; supervision.

## FUNDING INFORMATION

The study received no funding.

## CONFLICT OF INTEREST STATEMENT

The authors declare no potential conflict of interest for the present paper.

### PEER REVIEW

The peer review history for this article is available at https://www.webofscience.com/api/gateway/wos/peer‐review/10.1111/pai.70065.

## Supporting information


Table S1.

Table S2.
