# Supplementary material for: Anaphylaxis severity grade, during oral food challenges, assessed by five different classifications
Source: Pediatr Allergy Immunol. 2025 Mar 21;36(3):e70065. doi: 10.1111/pai.70065 (PMC11926947; doi:10.1111/pai.70065)
Supplement: Supplementary file 1 — Table S1. [file PAI-36-e70065-s002.docx]

**Supplementary Table 1** – Different classifications used for the present study.

| **ICD-11**  **Adapted from Ring & Messmer^7^** | **CoFAR^8^** | **Dribin^9^** | **EAACI^10^** | **Blasowsky^11^** |
| --- | --- | --- | --- | --- |
|  |  |  |  |  |
| **GRADE 1**   - One system involved: not life threatening. | **GRADE 1**   - Reaction involving 1 of the following organ systems (mild symptoms): cutaneous (generalized pruritus, generalized urticaria, flushing, angioedema), upper respiratory (rhinitis, cough unrelated to laryngeal oedema or bronchospasm), conjunctival (injection/redness, itching, tearing), gastrointestinal (nausea, abdominal pain with no change in activity level, single episode of vomiting and/or single episode of diarrhea). | **GRADE 1**   - Any mild: skin, gastrointestinal, mucosal/angioedema. | **GRADE 1**   - Skin: sudden itching of eyes and nose, generalized pruritus, flushing, urticaria, angioedema - Gastrointestinal: oral pruritus, oral tingling, mild lip; swelling, nausea or emesis, mild abdominal pain - Respiratory: nasal congestion and/or sneezing, rhinorrhea, throat tightness, mild wheezing - Neurological: change in activity level plus anxiety - Cardiovascular: tachycardia. | **GRADE 1**  No-Progressing and only one system involved   - Skin: urticaria, pruritus, flushing, angioedema - Gastrointestinal: oral pruritus, oral tingling, nausea, drooling - Upper respiratory: nasal symptoms, throat pruritus.   If rapidly progressing or > 1 system involved   - Skin: urticaria, pruritus, flushing, angioedema - Gastrointestinal: oral pruritus, oral tingling, nausea, drooling - Upper respiratory: nasal symptoms, throat pruritus. |
| **GRADE 2**   - More than one system involved: not severe. | **GRADE 2**   - Reaction involving 2 or more of the following organ systems (mild symptoms): cutaneous (generalized pruritus, generalized urticaria, flushing, angioedema), upper respiratory (rhinitis, cough unrelated to laryngeal oedema or bronchospasm), conjunctival (injection/redness, itching, tearing), gastrointestinal (nausea, abdominal pain with no change in activity level, single episode of vomiting and/or single episode of diarrhea) - OR reaction involving at least 1 of the following organ systems (moderate symptoms): cutaneous (generalized pruritus, generalized urticaria, flushing, angioedema), upper respiratory (rhinitis, cough unrelated to laryngeal oedema or bronchospasm), conjunctival (injection/redness, itching, tearing), gastrointestinal (nausea, abdominal pain with change in activity level, 2 episodes of vomiting and/or diarrhea). | **GRADE 2**   - 2 or more mild, ANY moderate: skin, gastrointestinal, mucosal/angioedema. | **GRADE 2**   - Skin: any of above - Gastrointestinal: any of above, crampy abdominal pain, diarrhea, recurrent vomiting - Respiratory: any of above, hoarseness, barky cough, difficulty swallowing, stridor, dyspnea, moderate wheezing - Neurological: light headedness feeling of pending doom - Cardiovascular: as above. | **GRADE 2**   - Gastrointestinal: crampy abdominal pain, sudden and/or recurrent vomiting, diarrhea - Upper respiratory: feeling of difficult breathing, hoarseness - Lower respiratory: sudden repetitive cough, chest tightness, mild to moderate bronchospasm - Neurological: sudden change in behavior or activity level, presyncope. |
| **GRADE 3**   - More than one system involved: severe. | **GRADE 3**   - Reaction involving 1 or more of the following organ systems: lower respiratory (throat tightness, wheezing, chest   tightness, dyspnea, cough that responds to short-acting bronchodilator treatment, including IM epinephrine, with or without supplemental oxygen), gastrointestinal (severe abdominal pain, >2 episodes of vomiting and/or diarrhea). | **GRADE 3**   - ANY mild: cardiovascular, neurologic, respiratory. | **GRADE 3**   - Skin: any of above - Gastrointestinal: any of the above loss of bowel control - Respiratory: any of above, cyanosis or saturation < 92 %, respiratory arrest - Neurological: confusion, loss of consciousness - Cardiovascular: hypotension, and/or collapse, dysrhythmia, severe bradycardia and/or cardiac arrest*.* | **GRADE 3**   - Upper respiratory: upper airway angioedema, stridor - Lower respiratory: severe bronchospasm - Neurological: confusion, somnolence, feeling of impending doom - Cardiovascular: sudden relevant hypotension, pale ang floppy child, short episode of collapse, syncope. |
| **GRADE 4**   - Cardiac and/or respiratory arrest. | **GRADE 4**   - Life-threatening reaction involving 1 or more of the following organ systems with or without other symptoms listed in grades 1-3: lower respiratory (throat tightness with stridor, wheezing, chest tightness, dyspnea, or cough associated with a requirement for supplemental oxygen and refractoriness to short-acting bronchodilator treatment, including IM epinephrine), or respiratory compromise requiring mechanical support, cardiovascular reduced blood pressure with associated symptoms of end-organ dysfunction. | **GRADE 4**   - ANY moderate: cardiovascular, neurologic, respiratory - OR severe: mucosal/angioedema. |  | **GRADE 4**   - Lower respiratory: respiratory failure - Neurological: loss of consciousness - Cardiovascular: cardiovascular failure, cardiac arrest. |
|  | **GRADE 5**   - Death | **GRADE 5**   - ANY severe: cardiovascular, neurologic, respiratory. |  |  |
