# Supplementary material for: Anaphylaxis severity grade, during oral food challenges, assessed by five different classifications
Source: Pediatr Allergy Immunol. 2025 Mar 21;36(3):e70065. doi: 10.1111/pai.70065 (PMC11926947; doi:10.1111/pai.70065)
Supplement: Supplementary file 2 — Table S2. [file PAI-36-e70065-s001.docx]

**Supplementary Table 2** – Comparison of the value of severity grading in the different classifications, in the 143 anaphylactic patients. Cells in green are those for which the value is the same as for the ICD-11. If the value is lower, cells are blue; if higher, they are red.

| **Patients** | **ICD-11^7^** | **CoFAR^8^** | **Dribin^9^** | **EAACI^10^** | **Blazowski^11^** |  | **Patients** | **ICD-11^7^** | **CoFAR^8^** | **Dribin^9^** | **EAACI^10^** | **Blazowski^11^** |  | **Patients** | **ICD-11^7^** | **CoFAR^8^** | **Dribin^9^** | **EAACI^10^** | **Blazowski^11^** |
| --- | --- | --- | --- | --- | --- | --- | --- | --- | --- | --- | --- | --- | --- | --- | --- | --- | --- | --- | --- |
| **1** | 2 | 2 | 1 | 1 | 1 |  | **49** | 2 | 2 | 1 | 1 | 1 |  | **97** | 2 | 2 | 2 | 2 | 1 |
| **2** | 2 | 2 | 1 | 1 | 1 |  | **50** | 2 | 2 | 1 | 1 | 1 |  | **98** | 2 | 2 | 3 | 2 | 2 |
| **3** | 3 | 2 | 3 | 2 | 3 |  | **51** | 3 | 4 | 4 | 3 | 4 |  | **99** | 2 | 2 | 1 | 1 | 1 |
| **4** | 2 | 2 | 1 | 1 | 1 |  | **52** | 2 | 2 | 1 | 1 | 1 |  | **100** | 3 | 4 | 4 | 3 | 4 |
| **5** | 3 | 3 | 3 | 2 | 3 |  | **53** | 2 | 2 | 1 | 1 | 1 |  | **101** | 2 | 2 | 2 | 1 | 1 |
| **6** | 2 | 2 | 1 | 1 | 1 |  | **54** | 2 | 2 | 2 | 1 | 1 |  | **102** | 2 | 3 | 2 | 2 | 2 |
| **7** | 3 | 3 | 3 | 2 | 3 |  | **55** | 3 | 4 | 4 | 3 | 4 |  | **103** | 2 | 2 | 2 | 1 | 2 |
| **8** | 2 | 2 | 3 | 2 | 2 |  | **56** | 2 | 2 | 2 | 1 | 2 |  | **104** | 2 | 3 | 2 | 2 | 2 |
| **9** | 2 | 2 | 2 | 1 | 2 |  | **57** | 2 | 2 | 3 | 2 | 2 |  | **105** | 2 | 2 | 3 | 2 | 2 |
| **10** | 3 | 2 | 3 | 2 | 2 |  | **58** | 2 | 2 | 1 | 1 | 1 |  | **106** | 2 | 2 | 1 | 1 | 1 |
| **11** | 3 | 4 | 3 | 3 | 3 |  | **59** | 3 | 4 | 4 | 3 | 4 |  | **107** | 2 | 3 | 2 | 2 | 2 |
| **12** | 2 | 2 | 2 | 1 | 1 |  | **60** | 2 | 2 | 3 | 2 | 2 |  | **108** | 2 | 3 | 2 | 2 | 2 |
| **13** | 2 | 2 | 1 | 1 | 1 |  | **61** | 2 | 2 | 1 | 1 | 1 |  | **109** | 3 | 3 | 3 | 2 | 2 |
| **14** | 3 | 3 | 3 | 2 | 2 |  | **62** | 2 | 2 | 2 | 1 | 1 |  | **110** | 2 | 2 | 1 | 1 | 1 |
| **15** | 3 | 3 | 3 | 2 | 2 |  | **63** | 2 | 2 | 1 | 1 | 1 |  | **111** | 2 | 2 | 1 | 1 | 1 |
| **16** | 2 | 2 | 1 | 1 | 1 |  | **64** | 2 | 2 | 1 | 1 | 1 |  | **112** | 2 | 2 | 2 | 1 | 1 |
| **17** | 2 | 2 | 1 | 2 | 1 |  | **65** | 3 | 4 | 4 | 3 | 4 |  | **113** | 3 | 4 | 4 | 3 | 4 |
| **18** | 2 | 2 | 2 | 2 | 2 |  | **66** | 2 | 2 | 1 | 1 | 1 |  | **114** | 2 | 2 | 2 | 2 | 2 |
| **19** | 2 | 2 | 2 | 1 | 2 |  | **67** | 3 | 4 | 3 | 2 | 3 |  | **115** | 2 | 2 | 2 | 1 | 1 |
| **20** | 2 | 2 | 2 | 1 | 2 |  | **68** | 2 | 2 | 1 | 1 | 1 |  | **116** | 2 | 3 | 2 | 2 | 2 |
| **21** | 2 | 2 | 3 | 2 | 2 |  | **69** | 3 | 3 | 3 | 2 | 2 |  | **117** | 2 | 2 | 1 | 1 | 1 |
| **22** | 2 | 3 | 3 | 2 | 2 |  | **70** | 3 | 3 | 3 | 2 | 2 |  | **118** | 2 | 3 | 2 | 2 | 2 |
| **23** | 2 | 2 | 1 | 1 | 1 |  | **71** | 2 | 2 | 2 | 2 | 2 |  | **119** | 2 | 2 | 2 | 1 | 2 |
| **24** | 2 | 3 | 2 | 2 | 2 |  | **72** | 2 | 2 | 1 | 1 | 1 |  | **120** | 2 | 2 | 1 | 1 | 1 |
| **25** | 2 | 2 | 2 | 1 | 2 |  | **73** | 2 | 2 | 1 | 1 | 1 |  | **121** | 2 | 2 | 1 | 1 | 2 |
| **26** | 2 | 2 | 1 | 1 | 1 |  | **74** | 2 | 2 | 2 | 2 | 2 |  | **122** | 2 | 2 | 2 | 2 | 2 |
| **27** | 2 | 2 | 2 | 1 | 1 |  | **75** | 2 | 2 | 2 | 1 | 1 |  | **123** | 3 | 3 | 2 | 2 | 2 |
| **28** | 3 | 4 | 4 | 3 | 4 |  | **76** | 2 | 2 | 1 | 1 | 1 |  | **124** | 2 | 3 | 3 | 2 | 2 |
| **29** | 2 | 2 | 2 | 1 | 2 |  | **77** | 2 | 2 | 1 | 1 | 1 |  | **125** | 3 | 3 | 3 | 2 | 2 |
| **30** | 2 | 2 | 3 | 2 | 2 |  | **78** | 3 | 4 | 3 | 2 | 3 |  | **126** | 3 | 4 | 4 | 3 | 4 |
| **31** | 3 | 3 | 3 | 2 | 1 |  | **79** | 2 | 2 | 2 | 1 | 1 |  | **127** | 3 | 2 | 3 | 2 | 2 |
| **32** | 2 | 2 | 1 | 1 | 1 |  | **80** | 2 | 3 | 3 | 2 | 2 |  | **128** | 2 | 2 | 1 | 1 | 1 |
| **33** | 2 | 3 | 1 | 2 | 2 |  | **81** | 2 | 2 | 1 | 1 | 1 |  | **129** | 3 | 3 | 3 | 2 | 2 |
| **34** | 2 | 2 | 1 | 1 | 1 |  | **82** | 3 | 3 | 3 | 2 | 2 |  | **130** | 2 | 3 | 3 | 2 | 2 |
| **35** | 2 | 2 | 2 | 2 | 2 |  | **83** | 3 | 4 | 4 | 3 | 4 |  | **131** | 2 | 2 | 1 | 1 | 1 |
| **36** | 2 | 3 | 2 | 2 | 2 |  | **84** | 2 | 1 | 1 | 1 | 1 |  | **132** | 2 | 2 | 2 | 1 | 1 |
| **37** | 3 | 3 | 3 | 2 | 2 |  | **85** | 2 | 2 | 2 | 2 | 2 |  | **133** | 2 | 2 | 1 | 1 | 1 |
| **38** | 2 | 2 | 1 | 1 | 1 |  | **86** | 2 | 2 | 1 | 1 | 1 |  | **134** | 2 | 2 | 1 | 1 | 1 |
| **39** | 2 | 2 | 3 | 2 | 2 |  | **87** | 2 | 2 | 2 | 1 | 1 |  | **135** | 2 | 2 | 1 | 1 | 1 |
| **40** | 2 | 2 | 1 | 1 | 1 |  | **88** | 2 | 2 | 3 | 2 | 2 |  | **136** | 3 | 4 | 4 | 3 | 4 |
| **41** | 2 | 2 | 2 | 2 | 2 |  | **89** | 2 | 2 | 1 | 1 | 1 |  | **137** | 3 | 2 | 1 | 2 | 1 |
| **42** | 2 | 2 | 1 | 1 | 1 |  | **90** | 2 | 2 | 1 | 1 | 1 |  | **138** | 2 | 2 | 2 | 1 | 2 |
| **43** | 3 | 4 | 4 | 3 | 4 |  | **91** | 2 | 2 | 1 | 1 | 1 |  | **139** | 2 | 2 | 2 | 1 | 1 |
| **44** | 2 | 2 | 2 | 1 | 2 |  | **92** | 2 | 2 | 2 | 1 | 2 |  | **140** | 2 | 2 | 3 | 2 | 2 |
| **45** | 2 | 3 | 2 | 2 | 2 |  | **93** | 3 | 4 | 4 | 3 | 4 |  | **141** | 2 | 2 | 1 | 1 | 1 |
| **46** | 2 | 3 | 2 | 2 | 2 |  | **94** | 3 | 4 | 3 | 3 | 3 |  | **142** | 2 | 2 | 2 | 1 | 1 |
| **47** | 2 | 2 | 1 | 1 | 1 |  | **95** | 2 | 2 | 2 | 1 | 2 |  | **143** | 2 | 2 | 1 | 1 | 1 |
| **48** | 2 | 2 | 1 | 1 | 1 |  | **96** | 3 | 4 | 4 | 3 | 4 |  |  |  |  |  |  |  |
